# Supplementary material for: Marriage of black phosphorus and Cu2+ as effective photothermal agents for PET-guided combination cancer therapy
Source: Nat Commun. 2020 Jun 8;11:2778. doi: 10.1038/s41467-020-16513-0 (PMC7280494; doi:10.1038/s41467-020-16513-0)
Supplement: Supplementary file 1 — Supplementary Information [file 41467_2020_16513_MOESM1_ESM.pdf]

Supplementary Information for

Marriage of black phosphorus and Cu^{2+} as effective photothermal agents for PET-guided combination cancer therapy

Kuan Hu et al.

Supplementary methods

Confocal microscopy

Fluorescein isothiocyanate (FITC) conjugated PEG was used to modify BP@$Cu_{0.4}$, generating BP@$Cu_{0.4}$@PEG-FITC and BP@$Cu_{0.4}$@PEG(FITC)-RGD. The cellular uptake of BPNS species was investigated by confocal microscopy. For live-cell confocal imaging, 35 mm cell culture dishes (Corning® BioCoat™ Poly-D-Lysine 35 mm Coverslip Bottom TC-treated Dishes, Catalog No. 354077) were used. 1×10^4 B16F10 melanoma cells were seeded in the 35 mm dishes and cultured for 24 hours for attachment. Then the medium was removed, and the cells were washed three times with PBS. BPNS species (BPNS, BP@Cu@PEG-FITC, or BP@Cu@PEG(-FITC)-RGD) in the full cell culture medium supplemented with 10% FBS were added to each dish. The mass of BPNS is equal and is about 200 µg per dish. Then the dishes were incubated at 37 °C for 6 hours. After that, the medium was removed and the cells were thoroughly washed three times with PBS. Notably, to eliminate the BPNS nonspecifically attached to the cell membrane, 37 °C PBS supplemented with 5% ethanol can be used to rinse the cells. Before imaging, 2 µg mL^{-1} Hoechst 33258 in the medium was used to stain the cell nucleus for 20 min in dark. Confocal imaging was acquired using a Keyence all-in-one fluorescence microscope BZ-X700 system. The average fluorescence intensity from differently treated cells was quantified using the built-in program.

Caspase-3/7 assay

B16F10 cells were seeded in 24-well plates at a density of 10^5 cells per well to ensure ~50-70% confluency on the day of the experiment. This assay was performed according to the manufacturer's protocol for the CellEvent Caspase-3/7 Green Detection Reagent (Invitrogen, Catalog No. C10423). Briefly, before BPNS species treatment, the medium was replaced with fresh medium. Then, either vehicle or BPNS species was added to a desirable working concentration and incubated for 48 hours in a cell incubator (37 °C and 5% CO_2). NIR irradiation (808 nm, 1 w cm^{-2}, 2 min) was applied to all cells after incubation for 4 hours. Remove the media from the cells, then add CellEvent caspase-3/7 reagent (5 µM in complete medium) to the cells. Incubate the cells at 37 °C for at least 30 min. After that, image the cell directly without any treatment using the Keyence microscope. The excitation/emission for detection is 488/520 nm. The fluorescence positive cells were counted using the built-in program.

TMRE Mitochondria membrane potential assay

B16F10 cells were seeded in 24-well plates at a density of 10^5 cells per well to ensure ~50-70% confluency on the day of the experiment. This assay was performed according to the manufacturer's protocol for the TMRE Mitochondria Membrane Potential Assay Kit (Abcam, Catalog No. ab113852). Briefly, before BPNS species treatment, the medium was replaced with fresh medium. Then, either vehicle or BPNS species was added to a desirable working concentration and incubated for 48 hours in a cell incubator (37 °C and 5% CO_2). NIR irradiation (808 nm, 1 W cm^{-2}, 2 min) was applied to all cells after incubation for 4 hours. Remove the media from the cells, then add TMRE solution (200 µM in complete medium) to the cells. Incubate the cells at 37 °C for at least 30 min.

After that, gently aspirate the media and replace it with 0.2% BSA in PBS. Then image the cell using the Keyence microscope. The excitation/emission wavelengths for detection are 549/575 nm. For flow cytometry analysis, the cells after TMRE treatment were collected by trypsinization and washed by PBS. The cells were suspended in 0.2% BSA in PBS and were analyzed using the flow cytometer (FACSCalibur, BD).

APO-BrdU TUNEL assay

The APO-BrdU TUNEL kit was used to detect the apoptotic cells. This assay was performed according to the manufacturer's protocol for the APO-BrdU TUNEL Assay Kit (Invitrogen, Catalog No. A35125). This assay is separated into two parts: cell preparation and fixation; detection of apoptotic cells. B16F10 cells were seeded in 24-well plates at a density of 10^5 cells per well to ensure ~50-70% confluency on the day of the experiment. Before BPNS species treatment, the medium was replaced with fresh medium. Then, either vehicle or BPNS species was added to a desirable working concentration and incubated for 48 hours in a cell incubator (37°C and 5% CO_2). NIR irradiation (808 nm, 1 W cm^{-2}, 2 min) was applied to all cells after incubation for 4 hours. After induction of cell apoptosis, the cells were washed with PBS for three times and trypsinized. Collect the cells and suspend 1-2 $\times$ 10^6 cells in 0.5 mL of PBS. Fix the cells with 1% (w/v) paraformaldehyde in PBS for 15 min. Wash the cells with PBS and resuspended in PBS for further labeling. Incubate the cells in the DNA-labeling solution for 60 min at 37 °C. Shake the samples every 15 min to keep the cells in suspension. After labeling, the cells were washed with rinse buffer and were incubated with Alexa 488 dye-labeled anti-BrdU antibody for 30 min at room temperature in dark. At the end of incubation, labeling the nucleus with propidium/RNase A staining buffer for 30 min at room temperature in dark. Analyze the sample by flow cytometry (FACSCalibur, BD).

Vybrant Dyecycle analysis

This assay was used as a complementary to detect the cell cycle of cells by the Propidium Iodide Flow Cytometry Kit (Abcam, ab139418). This kit can be used to detect the cellular DNA content and cell cycle distribution in live cells. We performed the assay according to the manufacturer's protocol for the Vybrant DyeCycle Orange Stains (Invitrogen, Catalog No. A35005). Briefly, B16F10 cells were seeded at a density of 10^5 cells per well on 12-well plates and cultured overnight. The cells were co-incubated with different BPNS species for 48 h. NIR irradiation (808 nm, 1 W cm^{-2}, 2 min) was applied to all cells after incubation for 4 hours. After incubation, the B16F10 cells were harvested, washed, and resuspended in complete media. Then add Vybrant DyeCycle Orange stain to the cells with a concentration of 10 µM. Incubate the cells at 37 °C for 30 min in dark. Finally, analyze the cells on a flow cytometer (FACSCalibur, BD) using 488 nm excitation and orange emission. Use linear amplification for DNA content and use a low flow rate for acquisition.

Intracellular ROS Measurement

B16F10 cells were seeded in 24-well plates (1 × 10^5 cells per well) and inoculated for 24 h. Then, the cells were treated with PBS, BPNS (100 ppm BPNS), BP@$Cu_{0.4}$ (100 ppm BPNS), and BP@$Cu_{0.4}$@PEG-RGD (100 ppm

BPNS) for 24 hours. NIR light was applied to all cells after incubation for 4 hours. Finally, the cells were incubated with 1 μL DCFH-DA (10 μM) for 20 min, washed with PBS (10 mM, pH = 7.4), and observed by CLSM.

Radio-TLC

The stability of BP@64Cu@PEG-RGD in mouse serum was examined by radio-TLC, using aluminum foil-backed silica gel matrix strips as the stationary phase. The BP@^{64}Cu@PEG-RGD solution (3.7 MBq) was added to 90 μL of mouse serum (freshly prepared) and then incubated at 37 °C for a designated time. A drop of the sample (2 μL) was taken out and loaded at a designated origin and left to air dry, before eluting with freshly-prepared 50 mM EDTA solution (pH = 7.5) as the mobile phase, taking care that the sample origin was not immersed. Free ^{64}Cu ion was chelated by EDTA and moved with the solvent front, whereas ^{64}Cu-labeled particles remained at the origin. The TLC strips were scanned by the MARITA–the single trace radioactivity thin-layer-chromatography detector. The peaks in the resulted spectra were integrated and the fraction for the intact BP@^{64}Cu@PEG-RGD was read out automatically. The TLC strips were also imaged by the digital autoradiography systems (Fujifilm FLA-5100 scanner with Aida Image analysis software, ai4r Le Beaver Real-time Imaging).

In vivo toxicity

Sixty healthy male C57BL/6Jms mice (6 weeks old) were obtained from Japan SLC (Shizuoka, Japan). All animals received humane care, and the Animal Ethics Committee of the National Institute of Radiological Sciences approved all the animal experiments. All experiments were carried out according to the recommendations of the Committee for the Care and Use of Laboratory Animals, National Institute of Radiological Sciences. The mice were separated into four groups and were injected with different agents (group 1: 100 μL saline; group 2: 100 μL BPNS in saline; group 3: 100 μL BP@Cu@PEG in saline, and group 4: 100 μL BP@Cu@PEG-RGD in saline). For group 2-4, the mass of BPNS injected into each mouse is about 200 μg. All the injections were via the tail vein. Three mice in each group were randomly taken out for blood hematological analysis. The mice left in each group were subjected to serum biochemical assay. For hematological analysis, 5 μL fresh blood was collected from the tail at day 1, 3, 15, and 30 following injection. Then the blood was diluted into a special buffer for analysis. For serum biochemical analysis, the mice were killed at various time points after injection (1, 3, 15 and 28 days, three mice per group at each time point). About 0.8 mL of blood was collected from each mouse to conduct serum biochemistry assay in Fujifilm biochemical analyzer (DRI-CHEM NX700, Tokyo, Japan). The major organs (liver, spleen, kidney, heart, and lung) were harvested, fixed in 10% neutral buffered formalin, processed routinely into paraffin, sectioned at 5 μm, stained with hematoxylin and eosin, and examined by digital microscopy.

Supplementary Figures

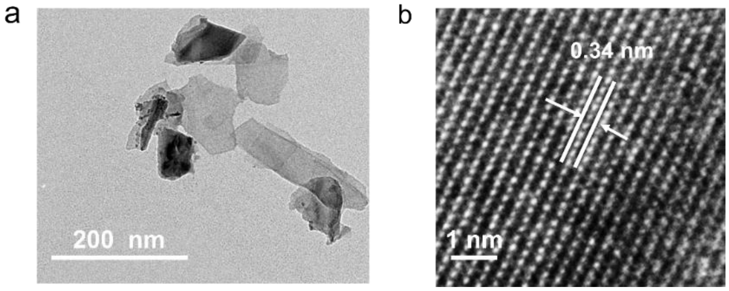

Supplementary Figure 1. TEM characterization. (A) TEM images of BPNS. (B) HR-TEM of BPNS and its lattice fringes. Similar TEM and HR-TEM images were obtained for three times experiments.

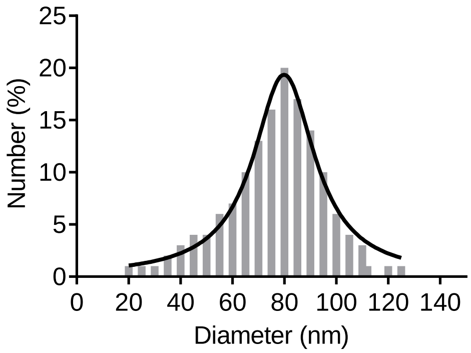

Supplementary Figure 2. AFM statistics of the lateral size of BPNS. The data are calculated based on three independent measurements.

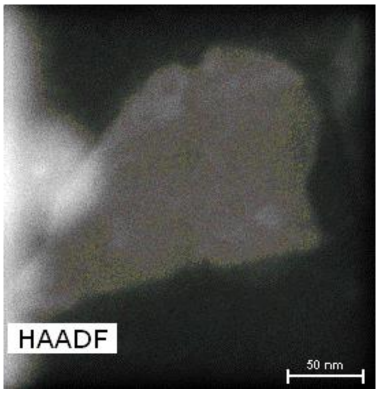

Supplementary Figure 3. STEM image of the BP@Cu corresponding to the elemental mapping in Figure 2d-e. Similar STEM images were observed for more than three independent experiments.

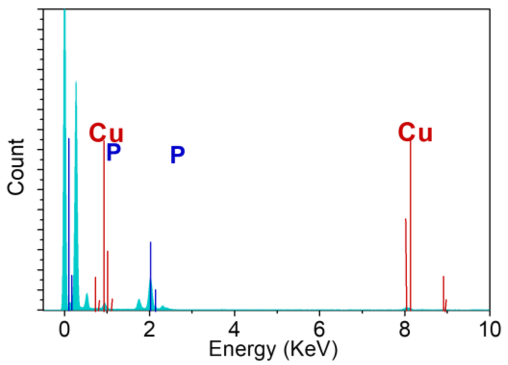

Supplementary Figure 4. Energy disperse X-ray spectroscopy analysis of the BPNS. Intense signals from Cu were observed in the spectrum.

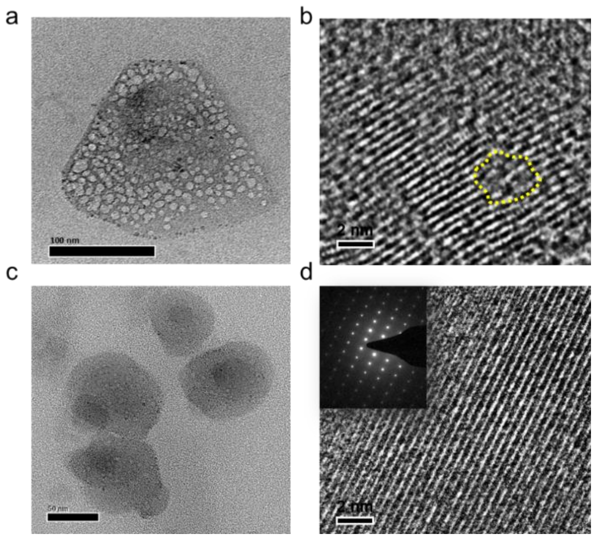

Supplementary Figure 5. TEM characterization. (a) TEM images of BP@$Cu_{0.4}$. (b) HR-TEM of BP@$Cu_{0.4}$. The yellow circle indicates the surface oxidation of BPNS. (c) TEM images of BP@$Cu_{0.4}$@PEG-RGD. (d) HR-TEM of BP@$Cu_{0.4}$@PEG-RGD. The inset in d shows the SAED pattern. Similar TEM and HR-TEM images were obtained for three times experiments.

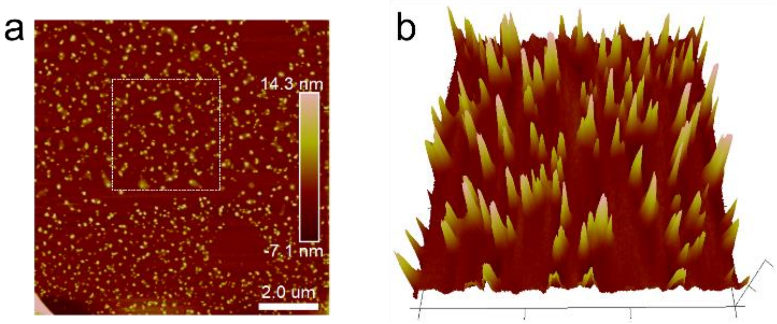

Supplementary Figure 6. AFM characterization. (a) AFM image of BP@Cu@PEG-RGD in a large area. The white frame denotes the selected area in Fig. 2g. (b) Three-dimensional morphology of BP@Cu@PEG-RGD. Similar AFM images were observed for more than two times experiments.

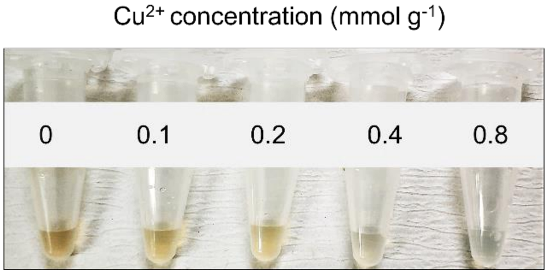

Supplementary Figure 7. The optical images of BPNS incubating with different concentrations of Cu^{2+} for 48 hours.

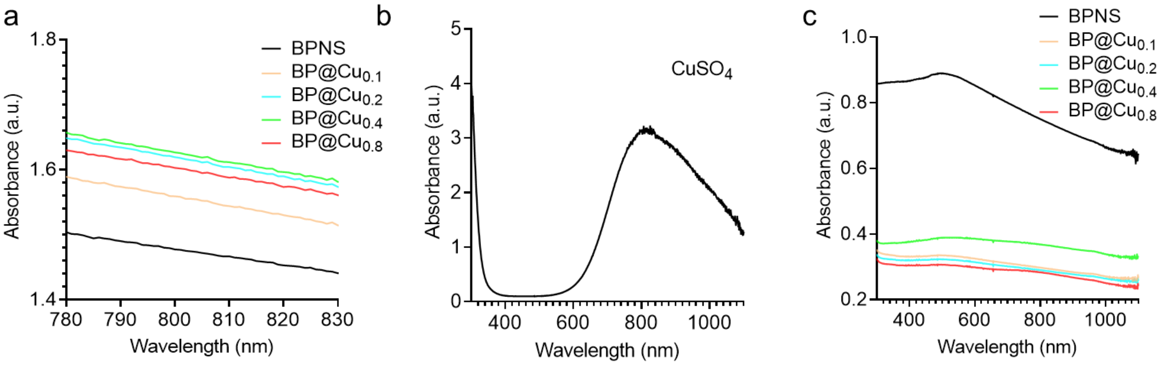

Supplementary Figure 8. UV-Vis-NIR characterization. (a) The absorbance spectra of BPNS and $BP@Cu_x$ in ddH_2O at the wavelength of 780-830 nm. (b) UV-Vis-NIR absorbance spectrum of 0.25 M $CuSO_4$ in double-distilled water (ddH_2O). (c) The absorbance spectra of BPNS and $BP@Cu_x$ in ddH_2O after 24 hours incubation at room temperature.

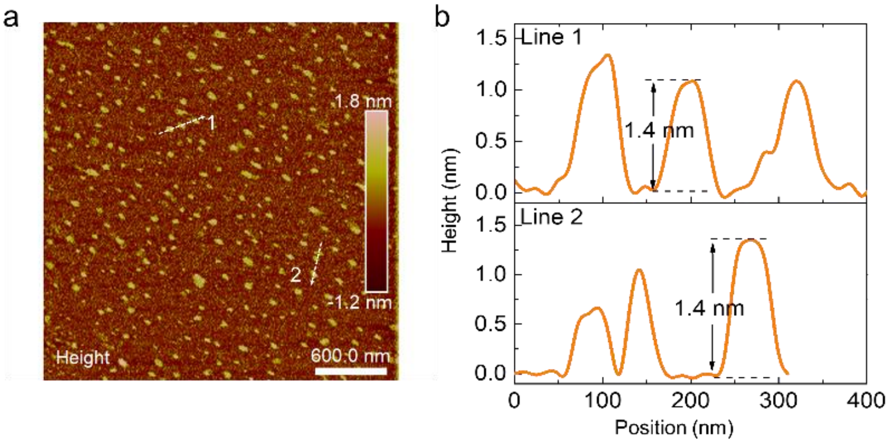

Supplementary Figure 9. AFM characterization. (a) AFM image of $BP@Cu_{0.8}$ after 24-hour incubation at room temperature. (b) AFM height profile of $BP@Cu_{0.8}$ along the two lines in image a.

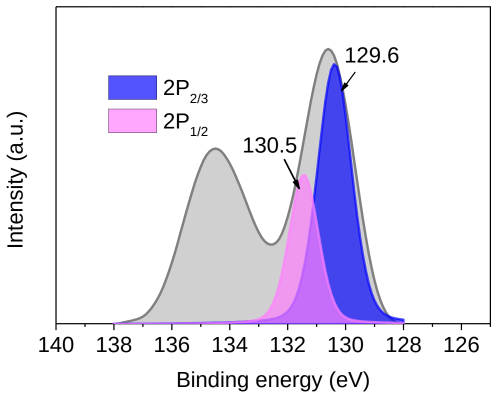

Supplementary Figure 10. High-resolution XPS P *2p* spectra of BPNS that was sprayed on cleaned silicon plates and placed in a normal atmosphere for 24 hours.

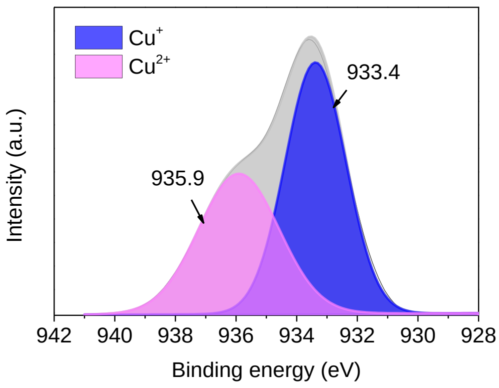

Supplementary Figure 11. High-resolution XPS P $2p_{2/3}$ spectra of Cu. The ratio of Cu^{+} and Cu^{2+} were simulated. The BPNS was sprayed on cleaned silicon plates and placed in a normal atmosphere for 24 hours.

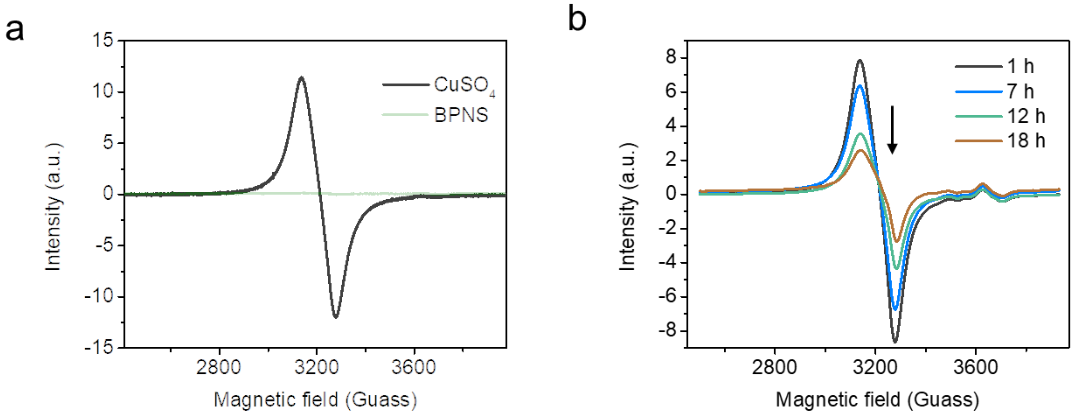

Supplementary Figure 12. EPR characterization. (a) EPR spectra of $CuSO_4$ and BPNS. (b) EPR spectra of BP@$Cu_{0.4}$ obtained at various time points to monitor the redox reaction between BPNS and Cu^{2+} at the atmosphere. The decrease in the intensity of Cu^{2+} indicates the reduction of Cu^{2+} to Cu^{+}.

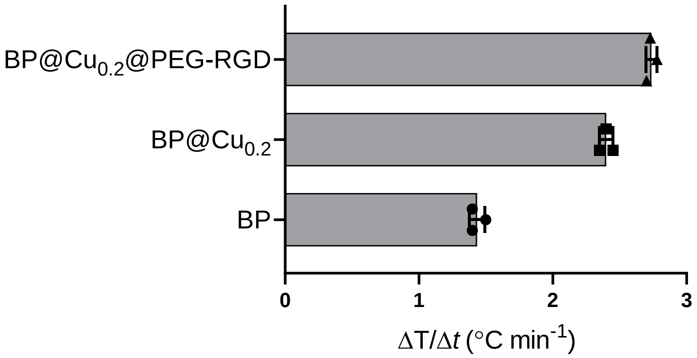

Supplementary Figure 13. The corresponding temperature increment per minute for BP, BP@Cu, and BP@Cu@PEG-RGD, respectively. The data represents the mean ± s.d., n = 3 independent experiments.

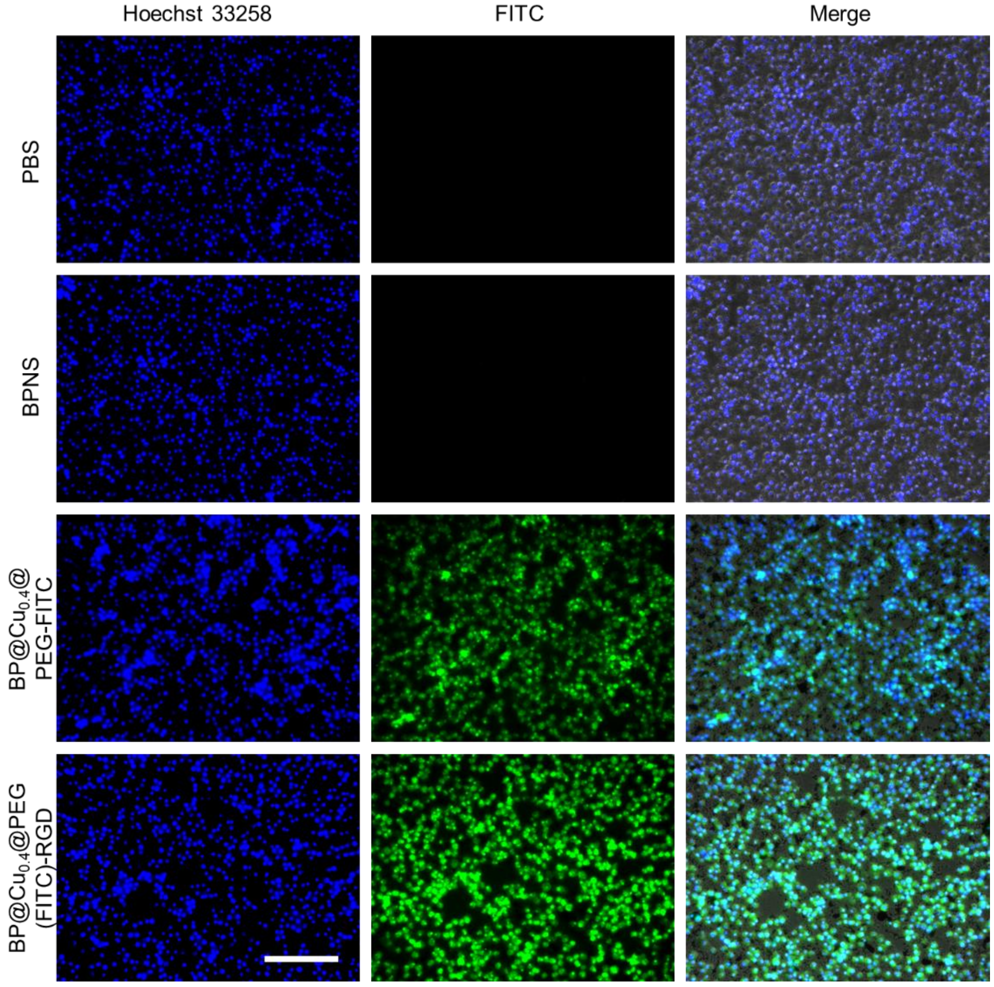

Supplementary Figure 14. Fluorescence images of live B16F10 cell incubating with PBS, BPNS (100 ppm), BP@$Cu_{0.4}$@PEG-FITC (100 ppm BPNS), or BP@$Cu_{0.4}$@PEG(-FITC)-RGD (100 ppm BPNS) for 6 hours. Cells were cultured in 35 mm live-cell imaging chamber overnight. Cells were pretreated with Hoechst 33258 (blue) for 10 min for nucleus staining before subjecting to confocal imaging. FITC-labeled PEG was used to modify BPNS. Scale bars, 400 μm for all panels. Similar results were observed for more than two times experiments.

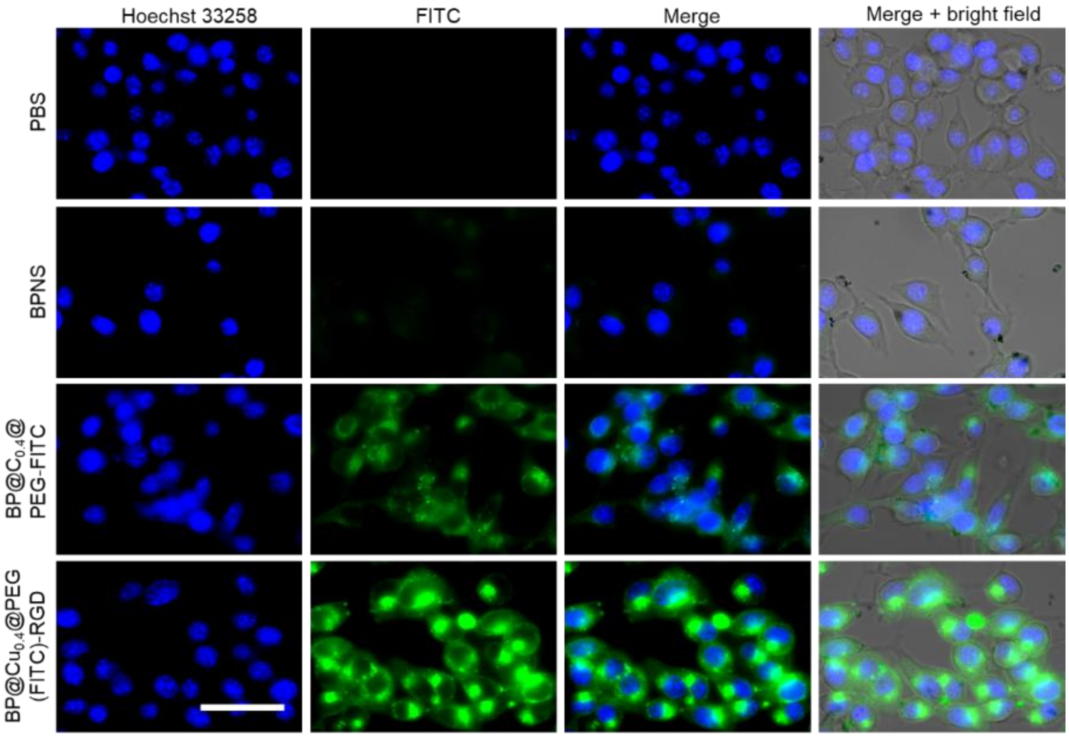

Supplementary Figure 15. Confocal images of live B16F10 cell incubating with PBS, BPNS (100 ppm), BP@$Cu_{0.4}$@PEG-FITC (100 ppm BPNS), or BP@$Cu_{0.4}$@PEG(-FITC)-RGD (100 ppm BPNS) for 6 hours. Cells were cultured in 35 mm live-cell imaging chamber overnight. Cells were pretreated with Hoechst 33258 (blue) for 10 min for nucleus staining before subjecting to confocal imaging. FITC-labeled PEG was used to modify BPNS. Scale bars, 50 μm for all panels. Similar results were observed for more than two times experiments.

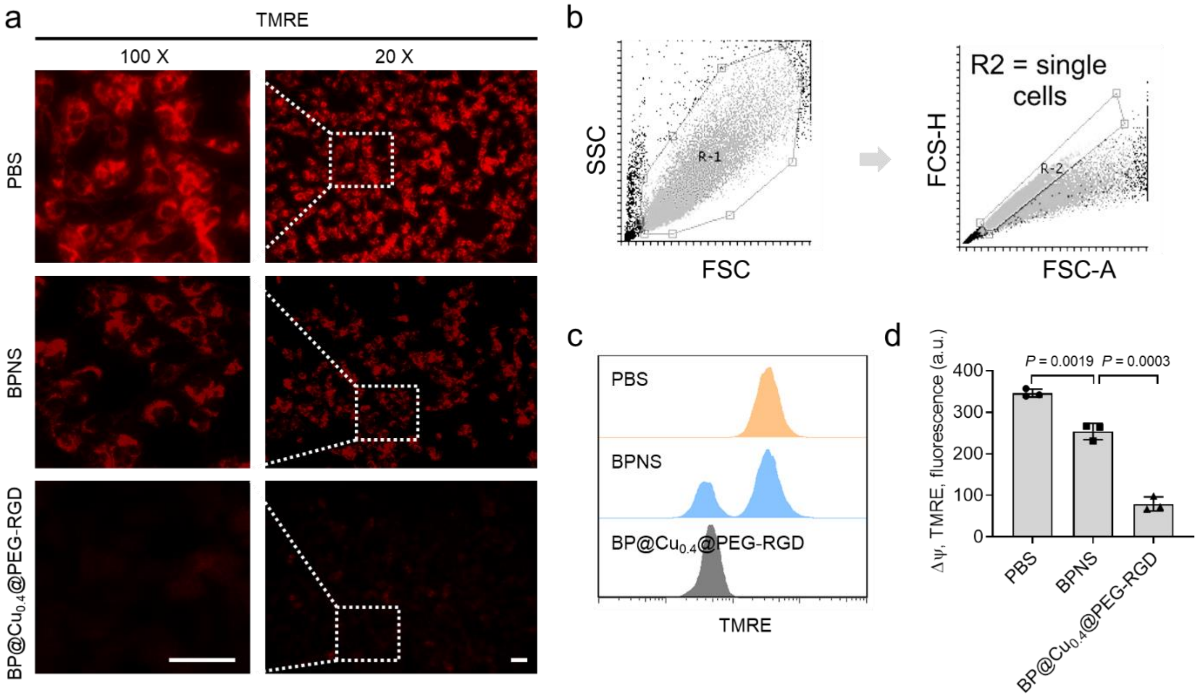

Supplementary Figure 16. TMRE assay. (a) Fluorescence microscopy imaging of TMRE-labeled mitochondria in live B16F10 cells treated with PBS, BPNS, or BP@$Cu_{0.4}$@PEG-RGD for 24 hours. Cells were treated with PBS, BPNS (100 ppm), BP@$Cu_{0.4}$ (100 ppm of BPNS), or BP@$Cu_{0.4}$@PEG-RGD (100 ppm of BPNS) for 48 hours. NIR irradiation (808 nm, 1 W cm^{-2}, 2 min) was applied to all cells after incubation for 4 hours. Cells after treatment were stained with 200 nM TMRE for 20 minutes in media, washed briefly with PBS and immediately imaged. The left arrow images show the enlarged view of selected areas (dashed box) in the right arrow. Scale bars, 50 µm for all panels. (b) Gating strategy used for the quantification of TMRE signal. The R1 region representing intact cells was used for single cell gating. The R2 region representing single cells was used for quantification of the TMRE signal. This gating strategy was identical in all FACS-related experiments in this study. (c) Analysis of cells by flow cytometry after TMRE staining. (d) Semi-quantification of membrane potential ($\Delta\Psi$) of cells based on the FACS data. Mean ± s.d., n = 3, the unpaired two-tailed student's t-test.

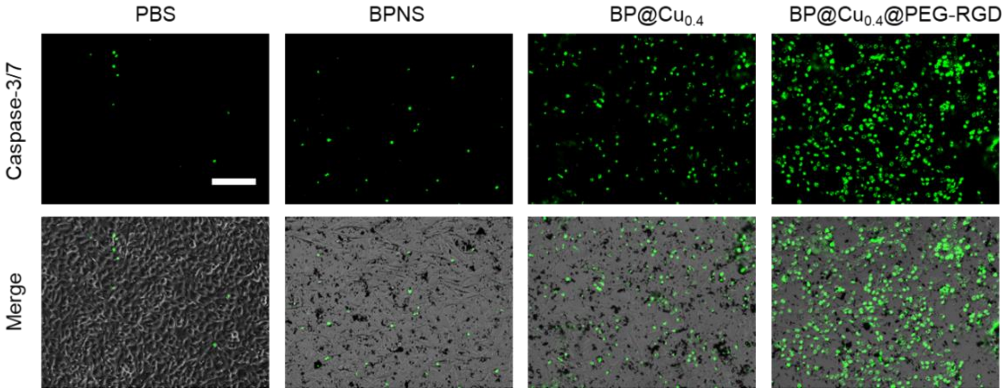

Supplementary Figure 17. Activation of caspase-3/7 in B16F10 cells after treatment with PBS, BPNS, BP@$Cu_{0.4}$, or BP@$Cu_{0.4}$@PEG-RGD for 24 hours. Cells were treated with PBS, BPNS (100 ppm), BP@$Cu_{0.4}$ (100 ppm of BPNS), or BP@$Cu_{0.4}$@PEG-RGD (100 ppm of BPNS) for 48 hours. NIR irradiation (808 nm, 1 w cm^{-2}, 2 min) was applied to all cells after incubation for 4 hours. Cells after treatment were loaded with 5 μM CellEvent Caspase-3/7 green detection reagent for 20 min and then imaged by fluorescence microscopy. Scale bars, 100 μm for all panels. Similar results were observed for more than two times experiments.

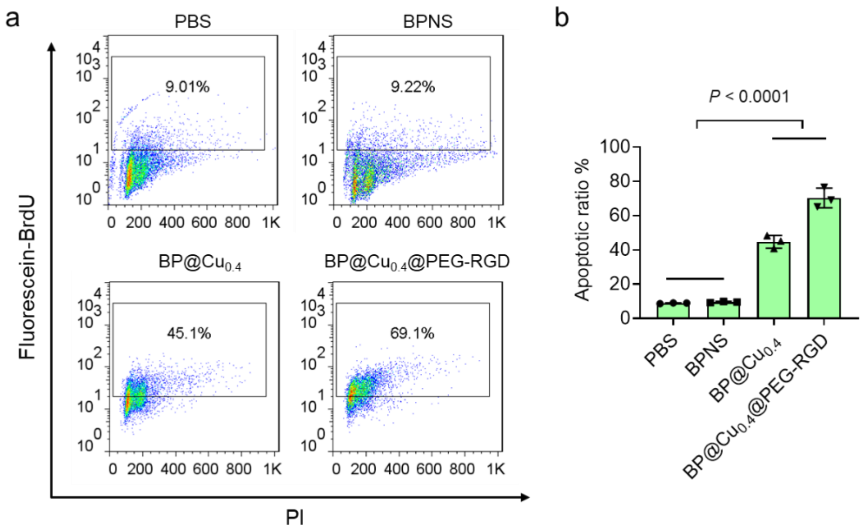

Supplementary Figure 18. Cells were treated with PBS, BPNS (100 ppm), BP@$Cu_{0.4}$ (100 ppm of BPNS), or BP@$Cu_{0.4}$@PEG-RGD (100 ppm of BPNS) for 48 hours. NIR irradiation (808 nm, 1 w cm^{-2}, 2 min) was applied to all cells after incubation for 4 hours. (a) Representative flow cytometry plots of Apo-BrdU staining of B16F10 cells treated with PBS, BPNS, BP@$Cu_{0.4}$, or BP@$Cu_{0.4}$@PEG-RGD for 24 hours. (b) Quantification of BrdU-positive cell. Mean ± s.d., $n = 3$, the unpaired two-tailed student's t-test. The error bars represent s.d. values.

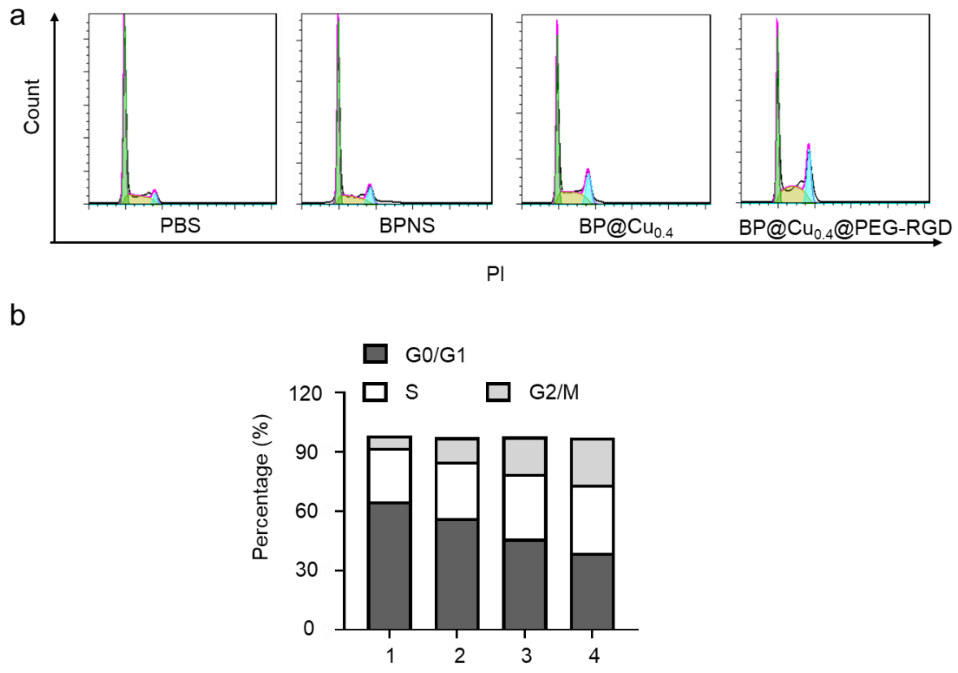

Supplementary Figure 19. Cells were treated with PBS, BPNS (100 ppm), BP@$Cu_{0.4}$ (100 ppm of BPNS), or BP@$Cu_{0.4}$@PEG-RGD (100 ppm of BPNS) for 24 hours. NIR irradiation (808 nm, 1 w cm^{-2}, 2 min) was applied to all cells after incubation for 4 hours. (a) Cell cycle distribution analysis of B16F10 cells stained with Vybrant Dyecycle Orange Stains and analyzed by flow cytometry. The data was processed by the Flowjo program. (b) Summary of cell percentage in each cell cycle phase, (1) PBS, (2) BPNS, (3) BP@$Cu_{0.4}$, (4) BP@$Cu_{0.4}$@PEG-RGD.

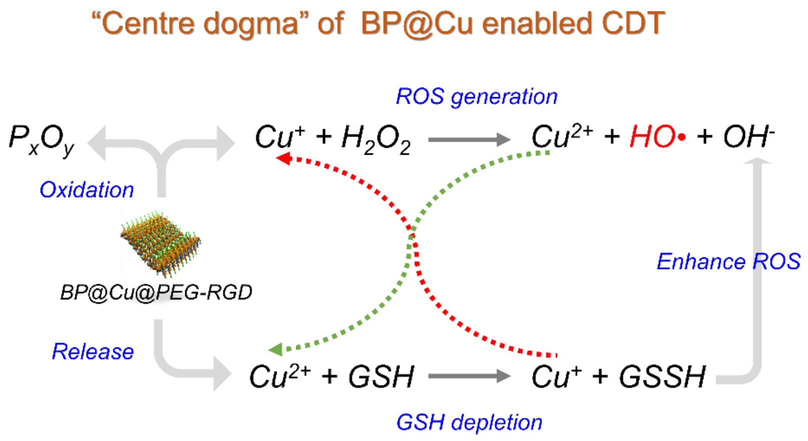

Supplementary Figure 20. Elucidation of the BP@Cu enabled CDT in the tumor microenvironment.

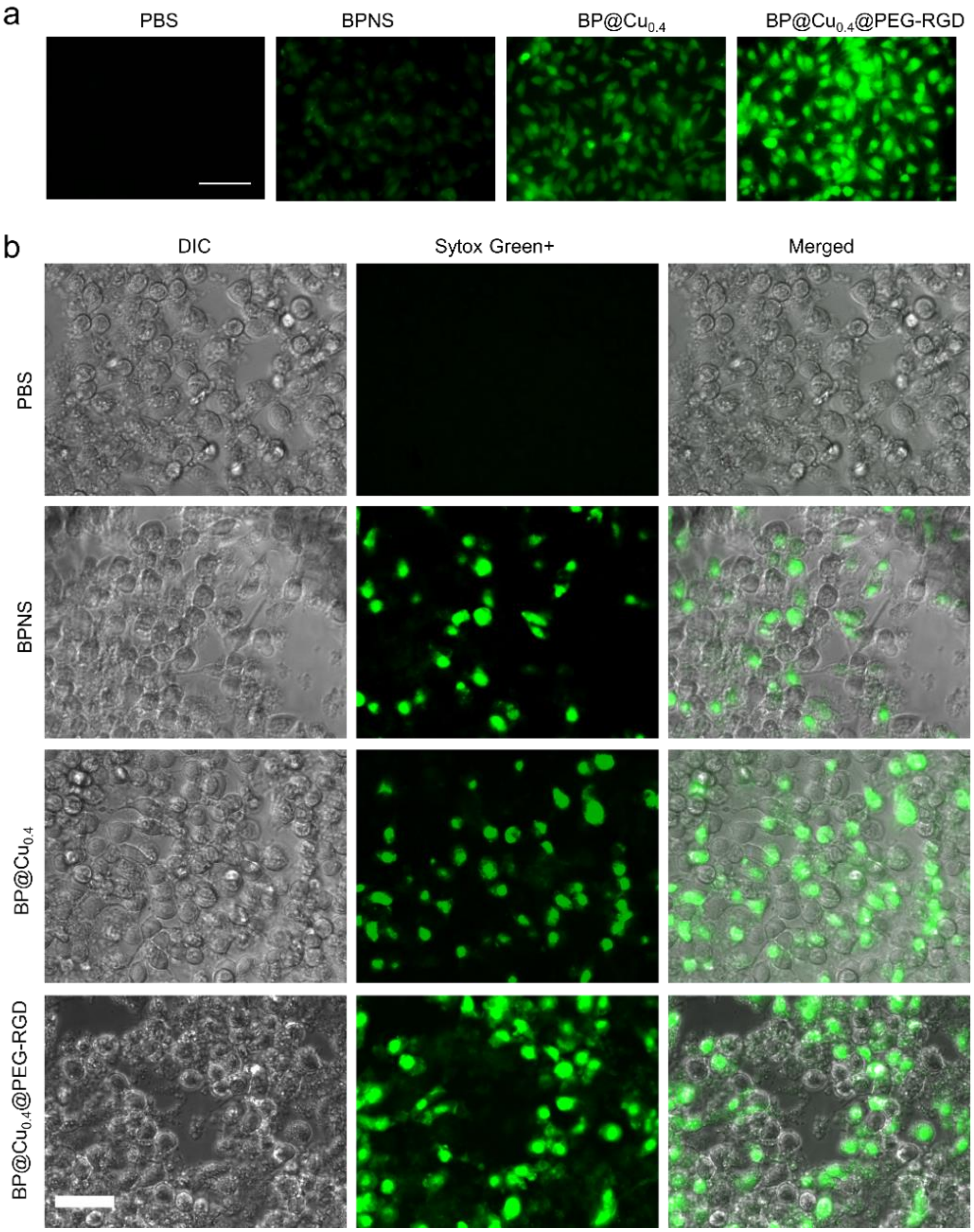

Supplementary Figure 21. DCFH-DA and Sytox green staining. (a) Measurement of intracellular ROS generation. Cells were treated with PBS, BPNS (100 ppm), BP@$Cu_{0.4}$ (100 ppm of BPNS), or BP@$Cu_{0.4}$@PEG-RGD (100 ppm of BPNS) for 24 hours. NIR irradiation (808 nm, 1 W cm^{-2}, 2 min) was applied to all cells after incubation for 4 hours. Cells after treatment were loaded with 10 µM DCFH-DA assay for 20 min and then imaged by fluorescence microscopy. Scale bars, 100 µm for all panels. (b) Sytox Green staining of B16F10 cells after treated with PBS, BPNS (100 ppm), and BP@$Cu_{0.4}$ (100 ppm of BPNS) or BP@$Cu_{0.4}$@PEG-RGD (100 ppm of BPNS), respectively. Cells were treated with the above-mentioned agents for 48 hours. NIR irradiation (808 nm, 1 W cm^{-2}, 2 min) was applied to all cells after incubation for 4 hours. Scale bars, 50 µm for all panels. Similar results were observed for more than two times experiments.

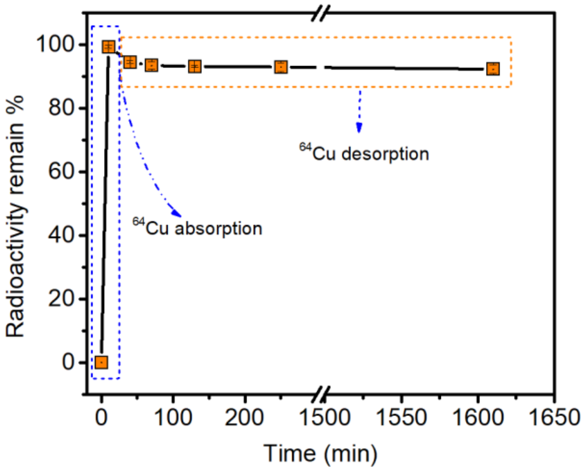

Supplementary Figure 22. The absorption and desorption of ^{64}Cu on BPNS. The first 10 min represents the absorption of ^{64}Cu by BPNS, and the remaining part shows the stability of BP@^{64}Cu in PBS. The radioactivity remain % = the radioactivity in the precipitate/the total radioactivity.

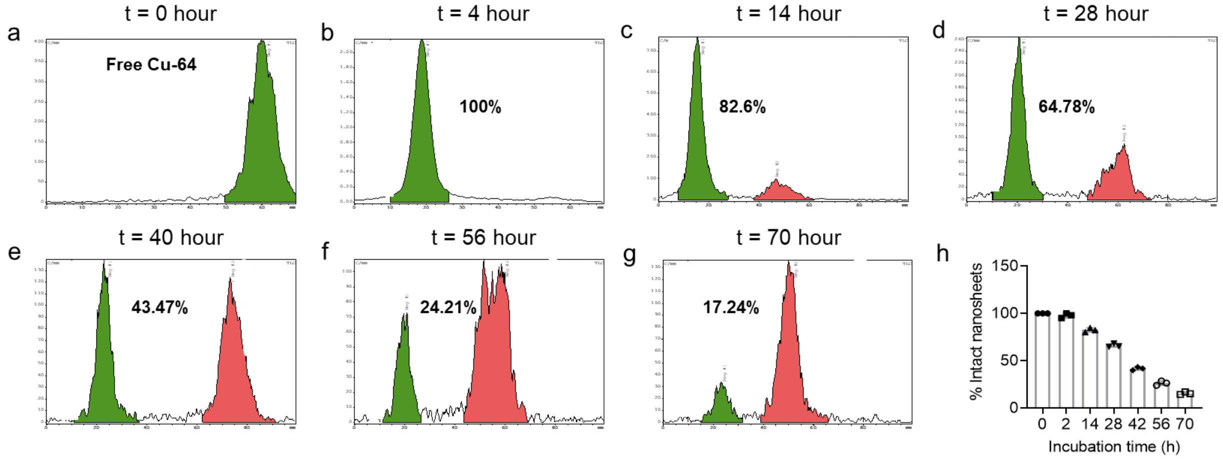

Supplementary Figure 23. (a) Radio-TLC chromatogram of free $^{64}Cu^{2+}$ ions in 0.2 M EDTA. (b-g) Stability data of BP@^{64}Cu@PEG-RGD in mouse serum monitored by radio-TLC chromatogram of at various time points. The radioactivity at different peaks was integrated. The fraction of radioactivity from the intact BP@^{64}Cu@PEG-RGD marked in green color was shown in each figure. (h) The percentage of intact nanosheets after different incubation periods. The data represent mean ± s.d., Error bars indicate standard deviation, *n* = 3 independent experiments.

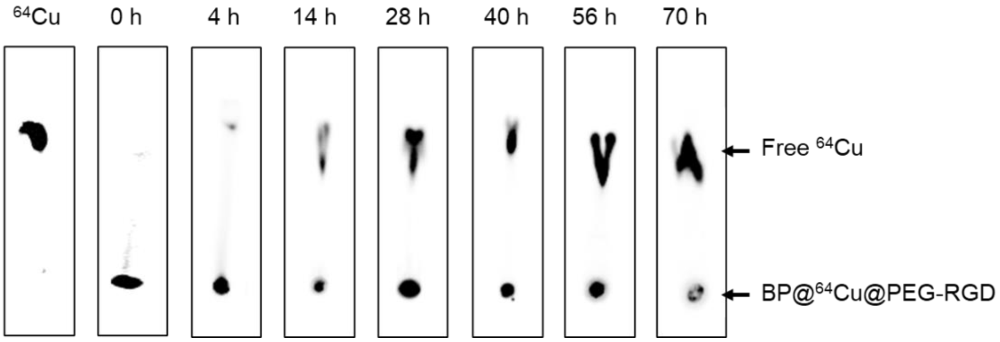

Supplementary Figure 24. Thin-layer chromatography (TLC) plates of BP@^{64}Cu@PEG-RGD at various time points when incubating in mouse serum. The images were recorded by an autoradiography machine. Free $^{64}Cu^{2+}$ ions were used as control. Similar results were observed for more than two times experiments.

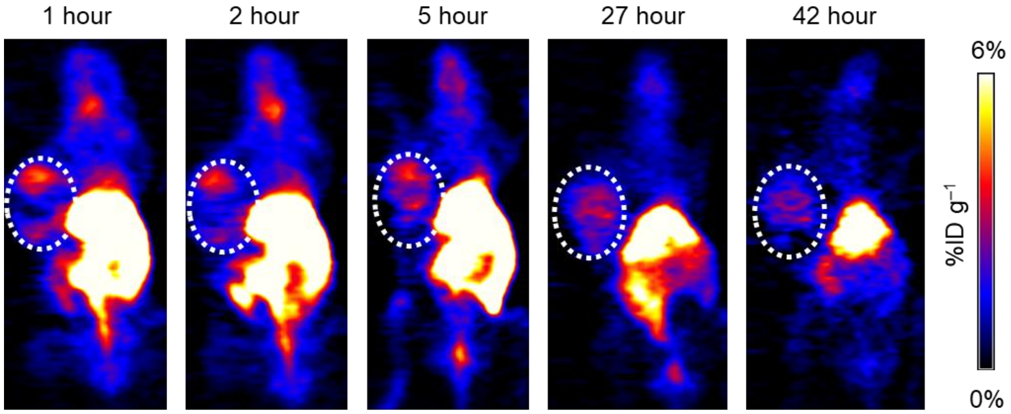

Supplementary Figure 25. MIP PET images of B16F10 tumor-bearing mice at 1, 2, 5, 27, and 42 hours after intravenous injection of free $^{64}Cu^{2+}$. The white circles denote the tumor sites.

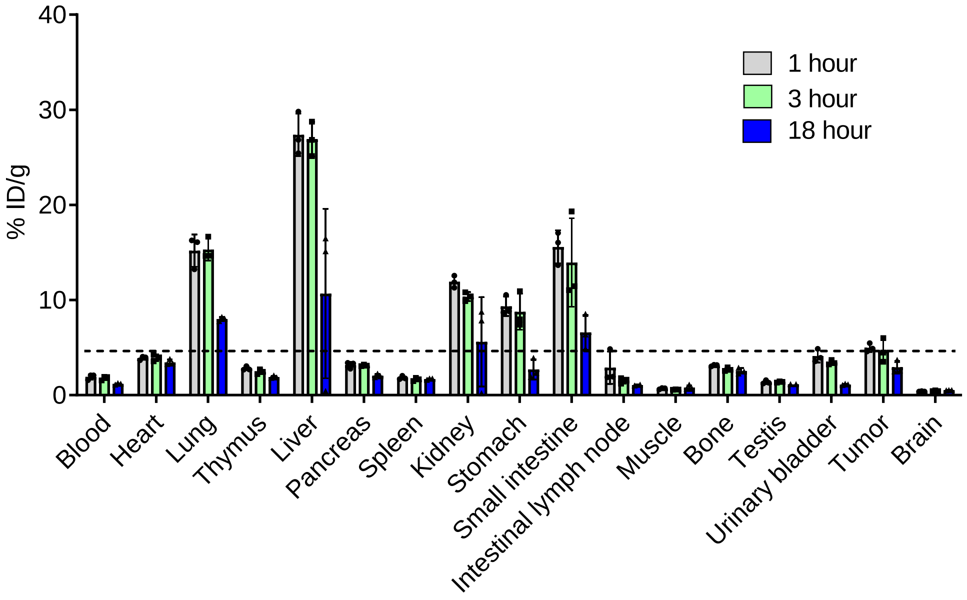

Supplementary Figure 26. *Ex vivo* biodistribution of free $^{64}Cu^{2+}$ ions in tumor and major organs of mice bearing B16F10 tumor at 1, 3, and 18 hours postinjection. Each point corresponds to mean ± s.d., *n* = 3. The error bars represent s.d. values.

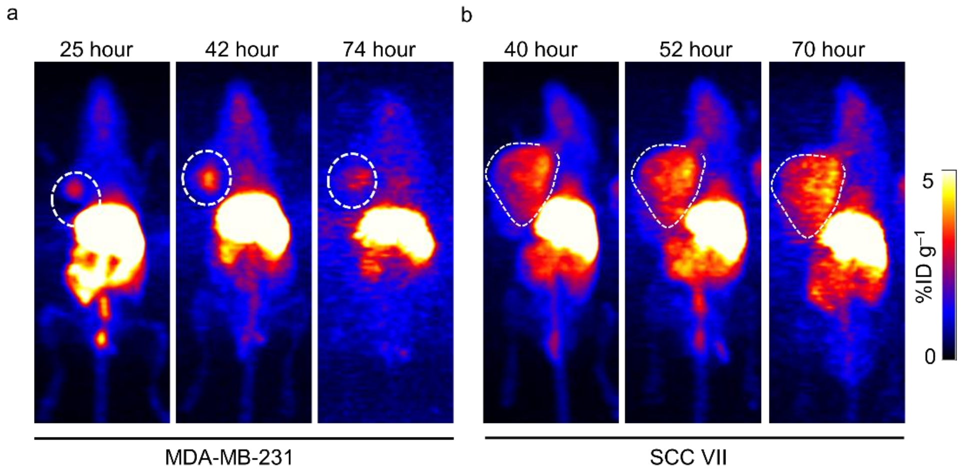

Supplementary Figure 27. Series MIP PET images of mice inoculated with MDA-MB-231 (Balb/c nude, a) and SCC VII (C3H/HeJ, b) tumor. The mice were intravenously injected with 11-17 MBq BP@^{64}Cu@PEG-RGD. Representative images are shown from 3 independent experiments (n = 3).

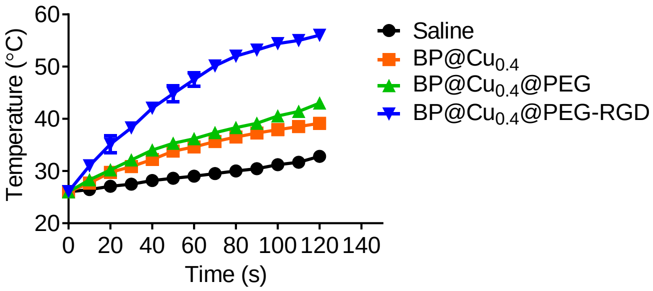

Supplementary Figure 28. Tumor temperature changes of mice bearing B16F10 tumors during laser irradiation as indicated in Fig. 7b. The data represents the mean ± s.d., n = 3 independent experiments.

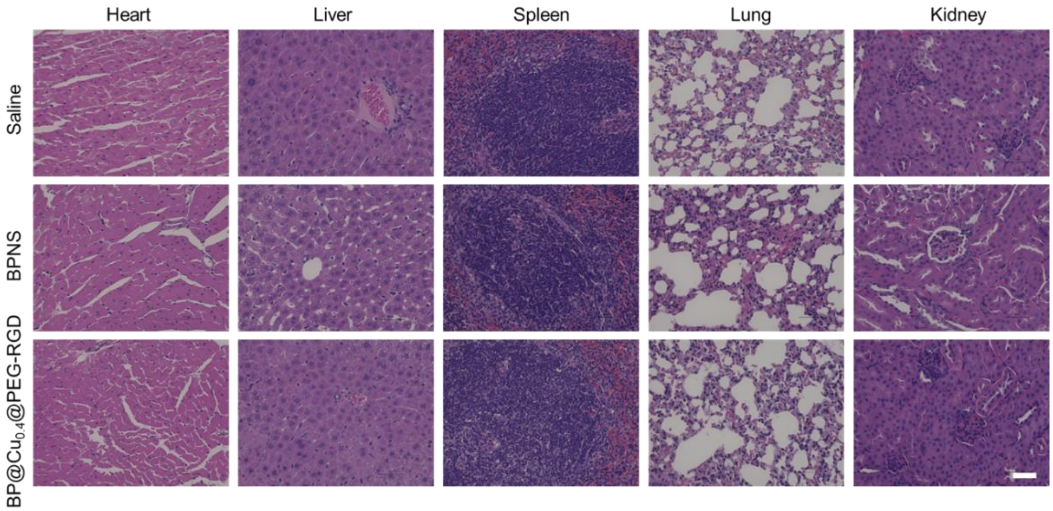

Supplementary Figure 29. Histology evaluation (hematoxylin and eosin-stained images) of the major organs (heart, liver, spleen, lung, and kidney) collected from mice treated with saline, BPNS, or BP@$Cu_{0.4}$@PEG-RGD at 15 days post-injection. Scale bars, 50 µm for all panels. Representative images are shown from two independent experiments.

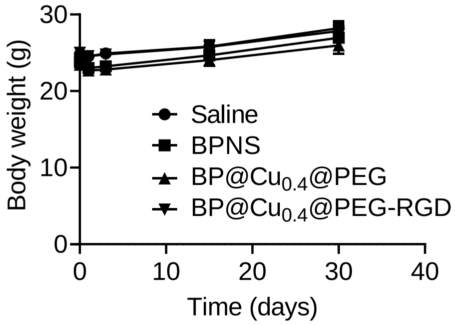

Supplementary Figure 30. Bodyweight of the mice intravenously injected with (1) saline, (2) BPNS, (3) BP@$Cu_{0.4}$@PEG, and (4) BP@$Cu_{0.4}$@PEG-RGD at 1, 3, 15, and 30 days post-injection. The administration dose is 200 µg per mouse according to the mass of BPNS. The results show the mean and s.d. (n = 3). The error bars represent s.d. values.

a. Day 1

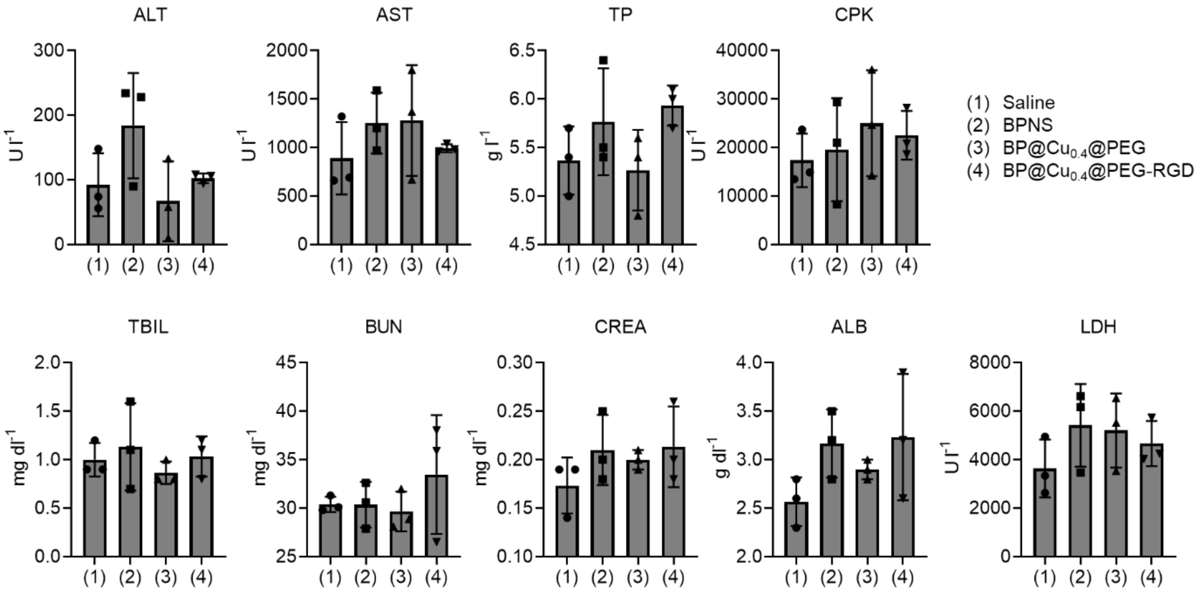
ALT
AST
TP
CPK
TBIL
BUN
CREA
ALB
LDH
U l⁻¹
g l⁻¹
mg dl⁻¹
g dl⁻¹
(1) (2) (3) (4)
(1) Saline
(2) BPNS
(3) BP@Cu0.4@PEG
(4) BP@Cu0.4@PEG-RGD

b. Day 3

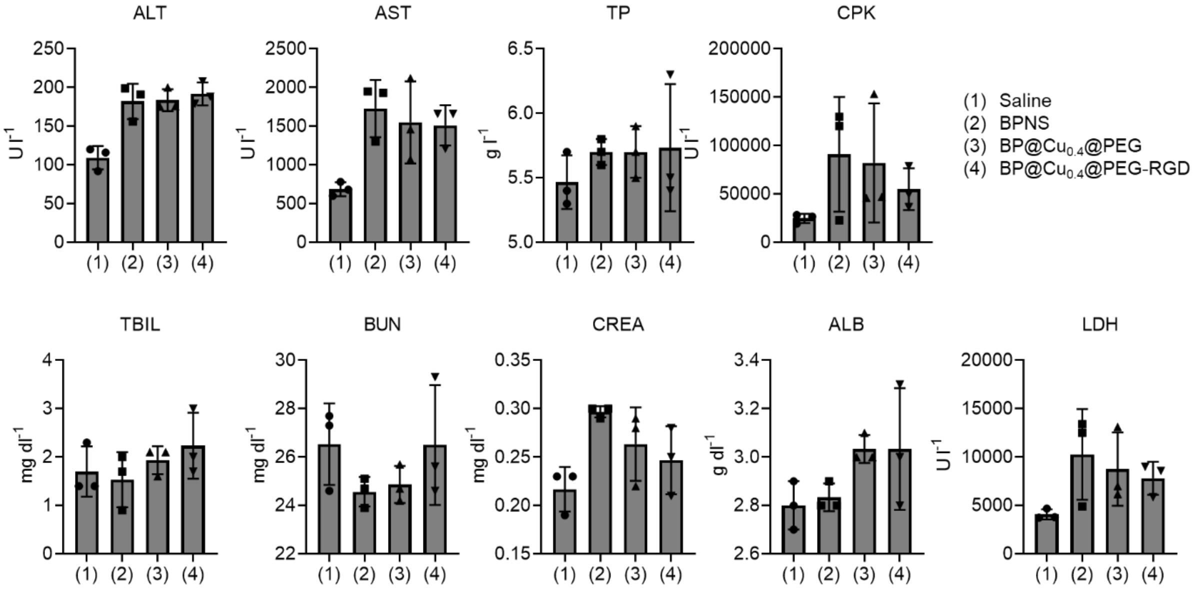
ALT
AST
TP
CPK
TBIL
BUN
CREA
ALB
LDH
U l⁻¹
g l⁻¹
mg dl⁻¹
g dl⁻¹
(1) (2) (3) (4)
(1) Saline
(2) BPNS
(3) BP@Cu0.4@PEG
(4) BP@Cu0.4@PEG-RGD

c. Day 15

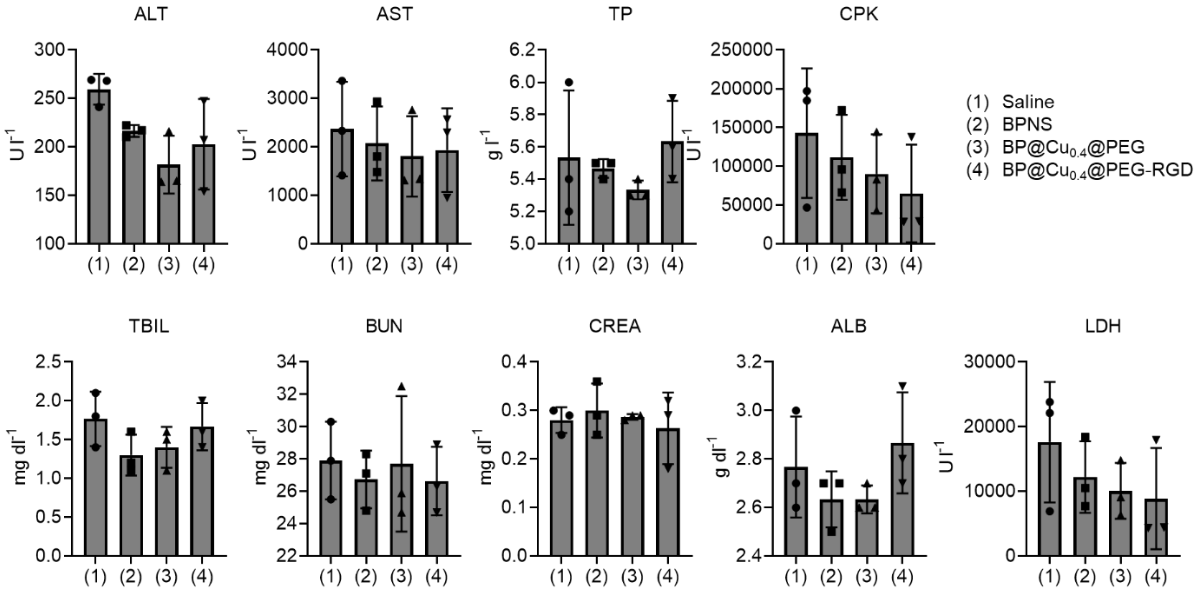

d. Day 30

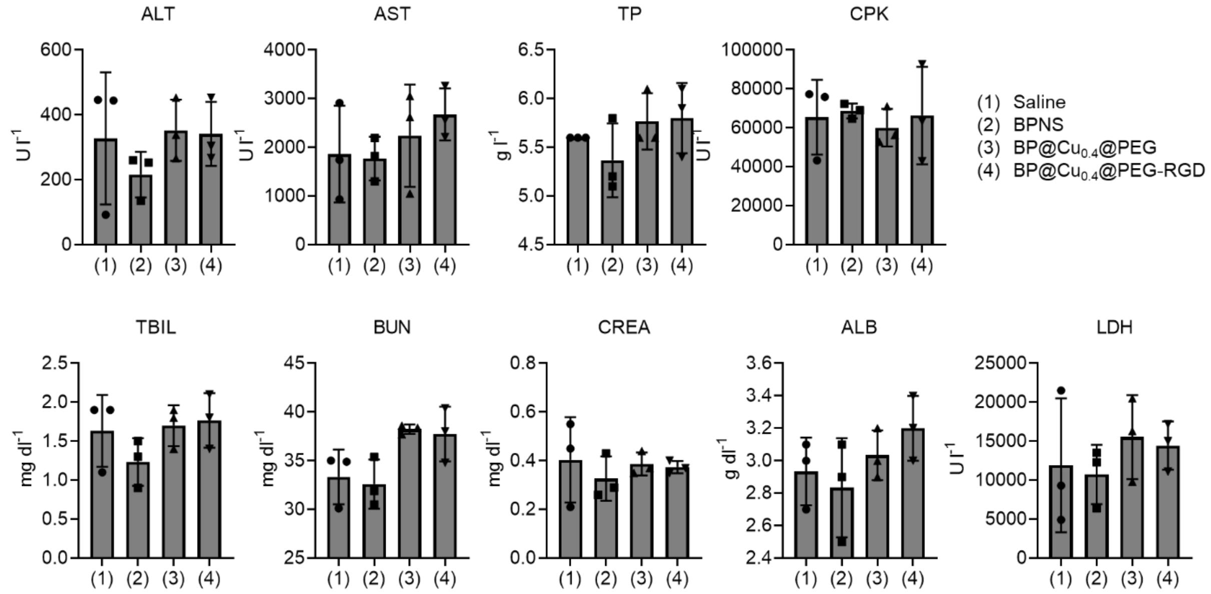

Supplementary Figure 31. Blood biochemical analysis of the C57/BL6J mice treated with (1) saline, (2) BPNS, (3) BP@$Cu_{0.4}$@PEG, and (4) BP@$Cu_{0.4}$@PEG-RGD at 1 (a), 3 (b), 15 (c), and 30 (d) days post-injection. The administration dose is 200 µg per mouse according to the mass of BPNS. The results show the mean ± s.d. (*n* = 3) of alanine aminotransferase (ALT), aspartate aminotransferase (AST), total protein (TP), creatine phosphokinase (CPK), total bilirubin (TBIL), blood urea nitrogen (BUN), creatinine (CREA), albumin (ALB), and lactate dehydrogenase (LDH). The error bars represent s.d. values.

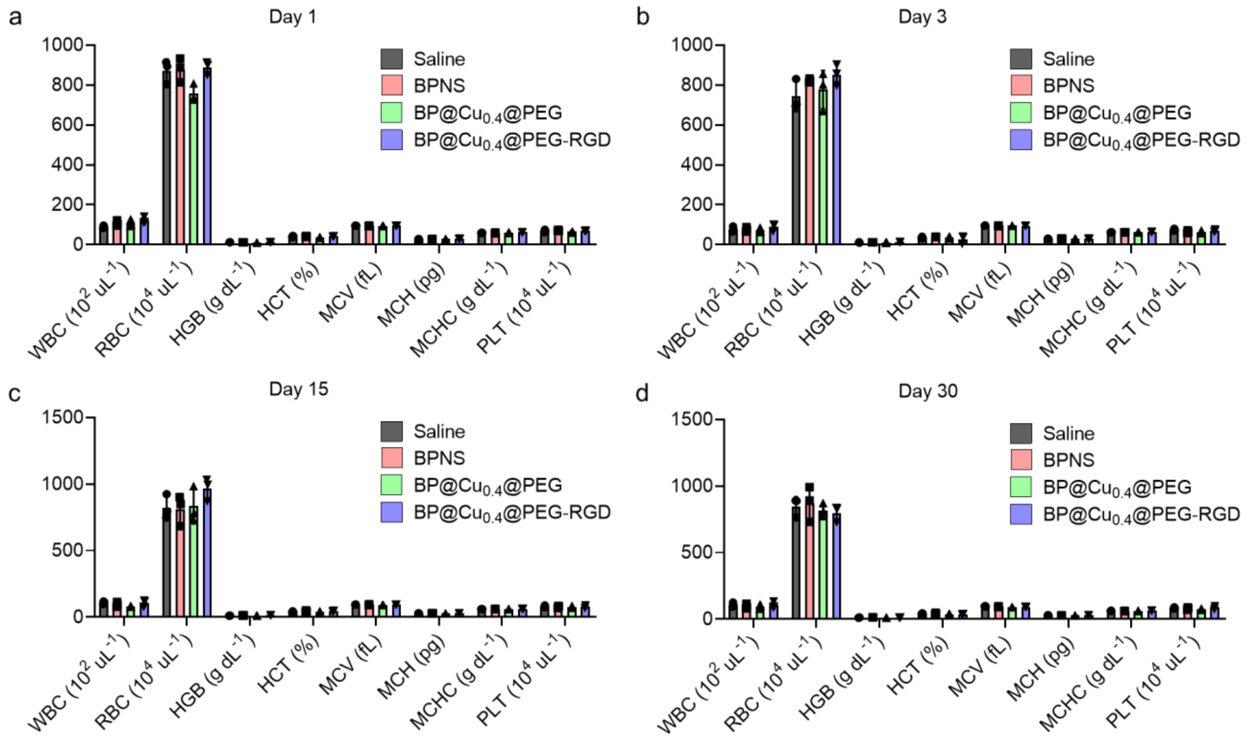

Supplementary Figure 32. Hematological data of the mice intravenously injected with (1) saline, (2) BPNS, (3) BP@$Cu_{0.4}$@PEG, and (4) BP@$Cu_{0.4}$@PEG-RGD at 1 (a), 3 (b), 15 (c), and 30 (d) days post-injection. The administration dose is 200 µg per mouse according to the mass of BPNS. The results show the mean ± s.d. (*n* = 3). The recording items are white blood cells (WBC), red blood cells (RBC), hemoglobin (HGB), hematocrit (HCT), mean corpuscular volume (MCV), mean corpuscular hemoglobin (MCH), mean corpuscular hemoglobin concentration (MCHC), and platelets (PLT). The error bars represent s.d. values.
